# HIV Patients’ Mindfulness Moderates How Death Anxiety and Depression Impact Life Quality

**DOI:** 10.1177/00302228241310072

**Published:** 2024-12-18

**Authors:** Chinenye Joseph Aliche, Erhabor Sunday Idemudia

**Affiliations:** 1 Faculty of Humanities, 274465 North West University, Potchefstroom, South Africa

**Keywords:** HIV patients, health-related quality of life, depression, mindfulness, death anxiety

## Abstract

Many people living with HIV (PLWH) experience death anxiety and depression, which may impact negatively on their health-related quality of life (HRQoL). Mindfulness is a psychological resource that protects against death anxiety and depression and promotes HRQoL. Although a direct association among these variables exist, little is known about their interactive effect. This study examined the role of PLWH mindfulness in the relationship between (a) death anxiety and HRQoL, and (b) depression and HRQoL. Participants included 311 HIV patients (199 women, 122 men, mean age = 30.52) selected from a tertiary healthcare institution in Nigeria. They completed relevant measures. Results showed that mindfulness moderated the effect of death anxiety on HRQoL. Mindfulness also buffered the effect of depression on HRQoL. Clinical efforts to improve HRQoL of PLWH should consider mindfulness-based training program due to its potentials in mitigating the effects of death anxiety and depression on health and recovery.

## Introduction

The diagnosis of HIV is an extremely stressful life event that may substantially impact on the health and quality of life of patients (Zhang et al., 2023). Despite the introduction of combined antiretroviral therapy, which has completely transformed HIV infection into a chronic disease (Yu et al., 2018), many people living with HIV (PLWH) are exposed to negative psychosocial consequences of HIV diagnosis including HIV-related stigma, depression, anxiety and poor health related quality of life (HRQoL) (Hoang et al., 2024; Zhang et al., 2023). HRQoL refers to how well a person functions in their life, and their perceived wellbeing across different domains including physical, relationship with others, cognitive, and treatment impacts (Hoang et al., 2024). The need to promote HRQoL of PLWH has led to the suggestion that HRQoL should be considered as an additional fourth pillar in the joint United Nations Programme on HIV/AIDS (UNAIDS)’s 90-90-90 targets for HIV testing, treatment and viral suppression (Safreed-Harmon, 2019). To that effect, it has become necessary that quality of life researchers focus on identifying those personality or individual differences factors that impact on HRQoL of PLWH. Death anxiety and depression have been identified as important psychosocial risk factors of HRQoL. Mindfulness, on the other hand, is a known psychosocial resource that protects people during stressful life challenges. Therefore, the goal of this study is to examine the role of HIV patients’ mindfulness in moderating how death anxiety and depression impacts life quality.

According to mindful coping model (Garland et al., 2011), an event is initially appraised as a threat that one is not capable of handling. Then the individual may initiate an adaptive response by decentering or withdrawing oneself from the initial stress appraisal, and then enter into the state of mindfulness, wherein the individual attends to the dynamic process of consciousness and experience broadened attention to the present moment. This state gives room for increased cognitive flexibility and heightened propensity to reappraise a stressful situation as meaningful or beneficial by reducing death-related anxiety and depressive symptoms; and activating positive emotional experiences such as optimism, hope and self-compassion, that promotes wellbeing and HRQoL. According to this theory, constantly engaging in mindful coping can consciously or sub-consciously increase trait mindfulness, which has a longitudinal effect, and can potentially buffer the impact of stressors (death anxiety and depression) on health outcome (Garland et al., 2011). Research revealed that higher mindfulness is associated with lower levels of death anxiety (Al-ghabeesh et al., 2024), and reduced symptoms of depression (Parmentier et al., 2019) and improved HRQoL (Gonzalez-Garcia et al., 2024), Additionally, levels of mindfulness is found to moderate the impact of anxiety on health outcome, such that anxiety was associated with poor health outcome only at low but not at high levels of mindfulness (Shen et al., 2023). Levels of mindfulness also moderated the relations of depressive symptomatology and health outcome, such that depressive symptoms were associated with poor mental health for people with low and average but not those with high levels of mindfulness (Hicke et al., 2018). These suggest that anxiety and depression can be risk factors of mental health outcome, and that increased mindfulness can buffer their adverse effect on wellbeing.

Indeed, mindfulness is one recent and promising approach for the promotion and maintenance of HRQoL of HIV patients (Gonzalez-Garcia et al., 2024). It involves essentially certain features such as observing, describing, responding with awareness, being nonjudgmental, and remaining nonreactive to daily experience (Gao et al., 2024). Mindfulness promotes a more balanced and less reactive approach to negative emotions associated with chronic health condition; and HIV patients with higher mindfulness tend to focus attention to what is going on right now, in an open, curious and greater acceptance attitude (Scott-Sheldon et al., 2019). Previous research has definitively established that mindfulness has a positive effect on wellbeing and quality of life among clinical and non-clinical samples (Aliche et al., 2024; Bhattarai et al., 2022; Gao et al., 2024). Moreover, there is robust evidence supporting the strong therapeutic effect of mindfulness-based therapies in promoting HRQoL of HIV patients (Gonzalez-Garcia et al., 2014; Samhkaniyan et al., 2015), indicating that mindfulness can serve as a protective factor ultimately resulting to improved wellbeing of PLWH.

Admittedly, the knowledge that one has been diagnosed with HIV may trigger feelings of uneasiness and uncertainty leading to existential crisis and death anxiety (Sherman et al., 2010). Additionally, many PLWH may believe that their life is meaningless; and may experience unhealthy emotional responses such as sadness, hopeless and depressive symptoms. Although these negative emotions associated with death anxiety and symptoms of depression may negatively impact on HRQoL of PLWH, the present study propose that greater mindfulness may buffer the impact of death anxiety, and depression, on HRQoL of HIV patients. Crucially, HIV patients with higher mindfulness may examine their thoughts and feelings without judgment, narrate their experiences in a non-evaluative manner, act with heightened awareness, and create an attitude of acceptance and non-reactivity. This strategy may help to reduce the avoidance and denial that are commonly linked with death anxiety and allow for a healthier processing of mortality related ideas. Mindfulness also serves to shift the focus away from past and future-oriented anxieties and towards present-moment sensations, lessening the cognitive and emotional load of death-related thoughts. Thus, individuals who cultivate a conscious approach to death may increase their psychological resilience, enjoy more emotional stability, and acquire a deeper sense of inner peace. These underscore the value of mindfulness as a potent strategy for reducing death anxiety and promoting general mental well-being (Al-ghabeesh et al., 2024). Furthermore, being mindful can help to decrease depressed mood and improve positive emotions. When individuals are connected to the present moment event with acceptance mindset, they may have greater ability to focus positively on how to solve the problem they are facing and the challenge itself, enabling them to disengage from negative emotions and avoid depressed mood. Even when negative emotions re-surface, mindfulness helps people to regulate emotions by diverting attention from event that causes negative emotion to positively rewarding ones (Parmentier et al., 2019).

## Literature Review

Death anxiety refers to an abnormal or persistent fear and worry about one’s own death (Tomás-Sábado et al., 2005). Among HIV patients, death anxiety is very common (Chukwuorji et al., 2020; Onu et al., 2021), with heightened death-related anxiety being associated with the development and maintenance of several mental health problems, including poor HRQoL (Onu et al., 2020; Zhao et al., 2022). However, some scholars argue that death anxiety is not related to poor HRQoL (Ezaka et al., 2022; Taghipour et al., 2017). Recent studies have suggested that those individuals whose HRQoL was not impacted by death anxiety may have been utilising other psychosocial resources such as meaning in life (Aliche et al., 2023b), and self-esteem (Zhao et al., 2022) in coping with their life challenges. Mindfulness is an adaptive coping strategy that may potentially influence the association of death anxiety and HRQoL due to its capacity to increase cognitive flexibility and decrease defensive responses to thoughts of death (Park & Pyszczynski, 2019).

However, depressive symptoms are also found among HIV patients (Liu et al., 2023; Moyo et al., 2023) and depressive symptoms are known to potentially lead to poor medication adherence, unwillingness to disclose HIV status (Yu et al., 2018), difficulty functioning optimally, increased perception of HIV-related stigma, and increased high-risk behaviours that fuel the transmission of HIV epidemics (Zhang et al., 2023). Depression could also lead to overall decrease in health care behaviours and poor HRQoL (Moyo et al., 2023; Zhang et al., 2023). However, recent research has found a positive association between depression and domains of HRQoL (Jerome et al., 2023). Thus, a psychological process such as mindfulness that has the capacity to facilitate positive reappraisal of a stressful life event can be very useful in decreasing anxiety and depressive symptoms among HIV patients (Garland et al., 2011; Shen et al., 2023).

Empirical evidence has supported the beneficial effect of mindfulness in reducing death anxiety (Al-ghabeesh et al., 2024; Askarizadeh et al., 2022) and depressive symptoms (Hicks et al., 2018; Parmentier et al., 2019; Xu et al., 2018). Likewise, mindfulness interventions decrease death related anxiety (Park & Pyszczynski, 2019; Xiang-Zi & Jia-Yuan, 2023) and symptoms of depression (Burnett-Zeigler et al., 2023; Musa et al., 2021). There is paucity of knowledge regarding the moderating effect of mindfulness in the relationship between death anxiety, depression and HRQoL among HIV patients. However, many researchers have identified mindfulness as a robust moderator of the effect of negative psychosocial antecedents on mental health outcome. For example, among college students, mindfulness moderated the relationship between anxiety and somatisation symptoms (Shen et al., 2023) and between trauma and anxiety symptoms (Tubbe et al., 2019). Among adolescence survivors of a traumatic event, mindfulness moderated the relationship between depression and posttraumatic growth (Xu et al., 2018); and depression and anxiety mediated the association between mindfulness and quality of life (Bergmann et al., 2021). In expectant parents, mindfulness moderated depression and quality of prenatal attachment (Hicks et al., 2018); and among students, mindfulness moderated the relationship between anxiety, uncertainty, and intercultural communication effectiveness (Nadeem & Koschmann, 2023). These studies suggest that mindfulness can modify the effect of stressors on health outcome.

## The Present Study

As far as it is known in literature, there is no current research exploring the relationship between death anxiety, depressive symptoms, mindfulness and HRQoL among PLWH, despite the recommendation by previous researchers on the need to examine the interactions among these variables (Aliche et al., 2023b; Xu et al., 2018; Zhao et al., 2022). Moreover, there is scarce literature on HIV and psychological health in the African region where the disease is endemic (Musa et al., 2021). This research is considered an important contribution to the global knowledge. A better understanding of the role of mindfulness in death anxiety, depression and quality of life could inform clinical efforts to address HIV-related outcomes. Thus, the current research aimed to examine the moderating role of mindfulness in the relationship between death anxiety, depressive symptoms and HRQoL among PLWH. Based on the literature, we hypothesized that among PLWH, mindfulness would moderate the relationship between HRQoL and (a) death anxiety, and (b) depressive symptoms (see Figure 1).

**Figure 1.**
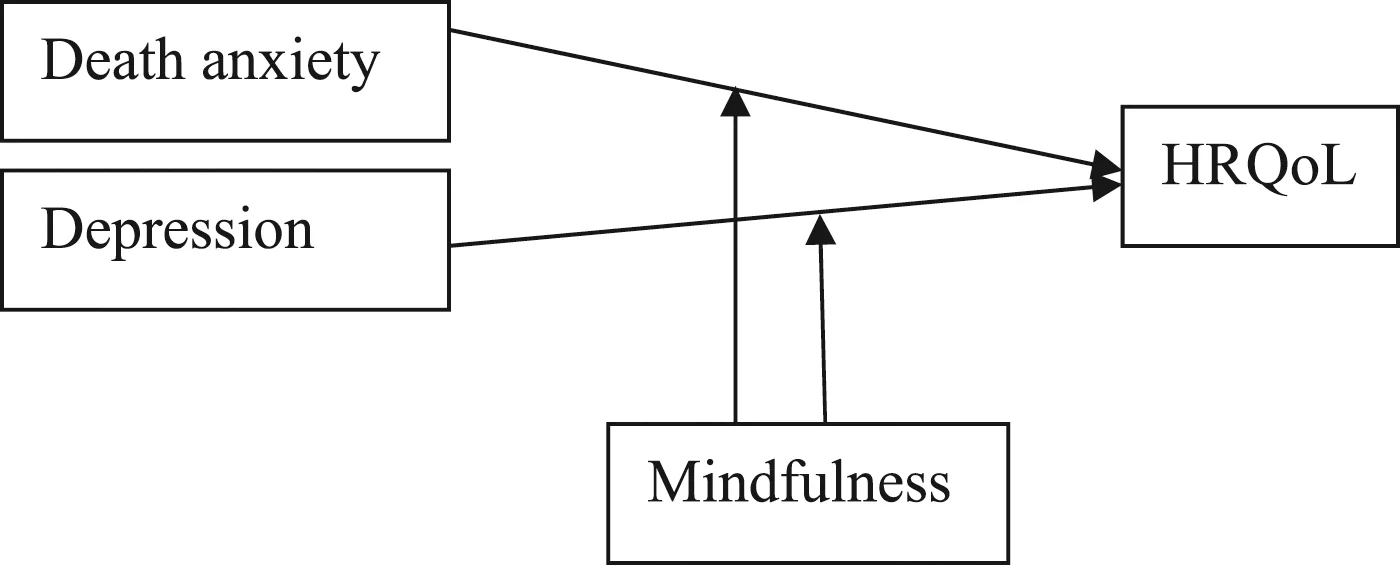
Conceptual model of mindfulness moderating the association of death anxiety, depression and HRQoL.

## Method

### Participants and Procedure

Participants consisted of 311 HIV positive patients drawn from the HIV/AIDS clinic of a tertiary healthcare institution in Nigeria. Inclusion criteria were (a) over 18 years of age (b) a confirmed HIV diagnosis for at least one year (c) at least secondary level formal education and (d) currently taking no antipsychotic medication. Of the 320 distributed survey, 311 were properly filled out and taken into analysis. The participants included 199 (64%) women, and 122 (36%) men. They ranged from age 21–61 years (Mean = 30.52, SD = 9.28). In terms of their marital status, 141 (45.3%) were married, 135 (43.4%) single, 12 (3.9%) divorced and 23 (7.4%) widow (er). Regarding their educational attainment, 166 (53.4%) had Secondary Education, 58 (18.6%) Ordinary National Diploma, 14 (4.5%) Higher National Diploma, 46 (14.8%) National Certification in Education, 24 (7.7%) Bachelors Degree and 3 (1%) had Master Degree.

Following the ethical approval from the research ethics committee of the institutional facility where the study was conducted (NHREC/55/02/07/2023), two trained research assistants approached participants at the hospital’s HIV and AIDS outpatient clinic while they were waiting to receive their antiretroviral drug treatment. The researchers explained the purpose of the study, confidentiality, the need to respond accurately to the items on the psychological instruments, and the right to withdraw at any time. After giving written informed consent, participants received the questionnaire and completed it there at the hospital. Data collection occurred from August 2023 to November 2023.

### Instruments

The Mindful Attention Awareness Scale (MAAS: Brown & Ryan, 2003) is 15 items (e.g., *I find it difficult to stay focused on what’s happening in the present*) scored on a 6-point scale from 1 = *Almost always* to 6 = *Almost never*. All items are aggregated to provide an overall mindfulness score from 15 to 90, with higher scores indicating greater mindfulness. The MAAS has been commonly used for measuring mindfulness, and has been validated among clinical samples, demonstrating good reliability exceeding 0.80 in all initial validation studies, and sensitivity to change. In the current study, estimate of Cronbach’s a = 0.79.

The Death Anxiety Inventory-Revised (DAI-R; Tomás-Sábado et al., 2005) consists of 17 items and four subscales: externally generated death anxiety, death acceptance, death finality and thoughts about death. The items (e.g., *‘I think I am more afraid of death than most people*”) are rated on a 5-point scale ranging from 1 = (*strongly disagree)*, to 5 = (*strongly agree)*. The total score ranges from 17 to 85 with higher score representing greater death anxiety. Developers reported that the scale is sufficiently valid and reliable Tomás-Sábado et al., 2005). In this study, death anxiety was measured using the DAI-R global score, and the estimate of Cronbach’s a = 0.81 was obtained.

The Iowa form (Kohout et al., 1993) which is a shorter version of the original 20 items Center for Epidemiological Studies Depression Scale (CES-D; Radloff, 1977) is 11 item (*“In the past week, I felt depressed*”, ‘*In the past week, I enjoyed life’* (reverse coded) scored on a 3-point scale from 1 = (*rarely or almost none of the time*) to 3 = (*almost or all of the time*). The total score range from 11 to 33 where higher scores signify greater depressive symptoms. Results revealed the scale has adequate validity and reliability indices (Kohout et al., 1993; Onyekachi et al., 2024). This study obtained a Cronbach’s a = 0.80.

The patient-reported outcome of quality of life - HIV (PROQoL; Duracinsky et al., 2012a; Duracinsky, et al., 2012b) consists of 38 items and four domains: physical symptoms, relationship with others, cognitive symptoms, and treatment impacts. The items (e.g., ‘*During the last two weeks, because I am HIV positive, I have felt restricted in my relationship with my family or friends’*) are scored on a 5-point scale from 0 = *(never),* to 4 = (*always)*. The procedure for scoring the PROQoL is reported elsewhere (Duracinsky et al., 2012a, 2012b), and the higher the scores, the better the HRQoL. The developers reported a minimum alpha reliability coefficient value of 0.77 across all domains. In this study, we obtained a reliability coefficient of 0.79.

### Data Analysis

The association between the demographic variables (e.g., age, gender), death anxiety, depression and HRQoL were calculated using Pearson correlation analysis. Then, the moderation analysis was calculated using Hayes PROCESS macro for SPSS Model 1 (Hayes, 2018). All analysis was carried out using the Statistical Packages for Social Sciences (SPSS), version 23.

## Results

Result in Table 1 showed that age and gender were not correlated with mindfulness, death anxiety, depression and HRQoL. Mindfulness was negatively correlated with death anxiety (r = −.16, *p* < .05), depression (r = −.52, *p* < .001), but positively correlated with HRQoL (r = .65, *p* < .001). Death anxiety was positively correlated with depression (r = .16, *p* < .01), and negatively correlated with HRQoL (r = −.16, *p* < .01). Depression and HRQoL were negatively correlated with each other (r = −.40, *p* < .001).

**Table 1.** Correlation of Demographic Variables, Predictors and Dependent Variables.

	Variables	1	2	3	4	5	Mean	SD
1	Age	—					30.52	9.28
2	Gender	.11	—				—	—
3	Mindfulness	.04	.05	—			61.77	11.52
4	Death anxiety	−.03	−.07	−.16^*^	—		52.68	20.24
5	Depression	−.11	−.02	−.52^***^	.16^**^	—	20.57	8.55
6	HRQoL	.08	.06	.65^***^	−.16^**^	−.40^***^	104.69	29.93

*Note.* ***p = .001, **p = .01, *p = .05; Gender was coded as male = 0, female = 1; HRQoL = Health-related quality of life.

### Moderation Results

The results of the moderation hypothesis are presented in Table 2 with death anxiety and depression considered as independent variables, mindfulness as a moderator variable, and HRQoL as the outcome variable. Result showed that death anxiety was not significantly associated with HRQoL (B = −.06, *p* = .383). Mindfulness was significantly and positively associated with HRQoL (B = 2.54, *p* = .000) suggesting that greater mindfulness was associated with improved HRQoL. The interaction between death anxiety and mindfulness on HRQoL was significant (B = .02, *p* = .035) indicating that mindfulness moderated the association between death anxiety and HRQoL. Examination of the interaction slope (Figure 2) revealed that death anxiety was associated with poor HRQoL only for HIV patients with low (B = −.20, *p* = .040), but not those with average (B = −.06, *p* = .383) and high (B = .05, *p* = .559) levels of mindfulness.

**Table 2.** Hayes PROCESS Results of Mindfulness Moderating the Relationship of Death Anxiety, Depression and HRQoL.

Variables	B	SE	T	*p*	95%CI
Mindfulness moderation of death anxiety and HRQoL					
Death anxiety (DA)	−.06	.06	−.87	.383	[-.18, .07]
Mindfulness (M)	2.54	.21	12.14	.000	[2.13, 2.95]
DA x M	.02	.01	2.11	.035	[.01, .04]
Mindfulness moderation of depression and HRQoL					
Depression (D)	−.28	.17	−1.66	.099	[-.61, .05]
Mindfulness (M)	2.13	.24	9.02	.000	[1.66, 2. 59]
D x M	.09	.03	3.61	.000	[.04, .14]

*Note.* HRQoL – health-related quality of life.

**Figure 2.**
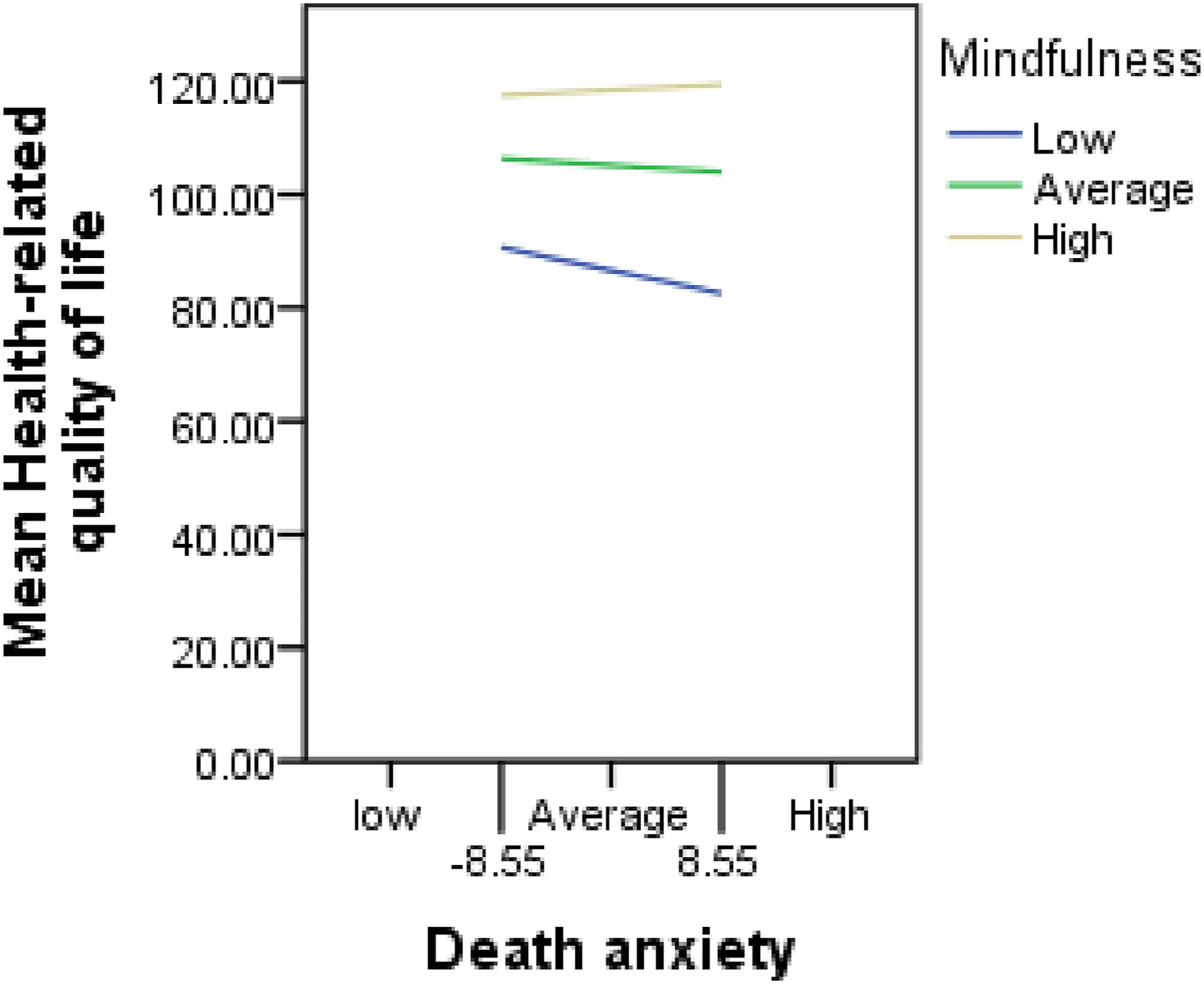
Showing the moderating role of mindfulness in the association of death anxiety and health-related quality of life.

When we examined the moderating role of mindfulness in the association of depression and HRQoL, results revealed that depression was not significantly associated with HRQoL (B = −.28, *p* = .099). Mindfulness was significantly and positively associated with HRQoL (B = 2.13, *p* = .000) and moderated the association between depression and HRQoL (B = .09, *p* = .000). The interaction slope (Figure 3) shows that depressive symptom was associated with poor HRQoL only for HIV patients with low (B = −.94, *p* = .000), but not those with average (B = −.28, *p* = .098) and high (B = .19, *p* = .381) levels of mindfulness.

**Figure 3.**
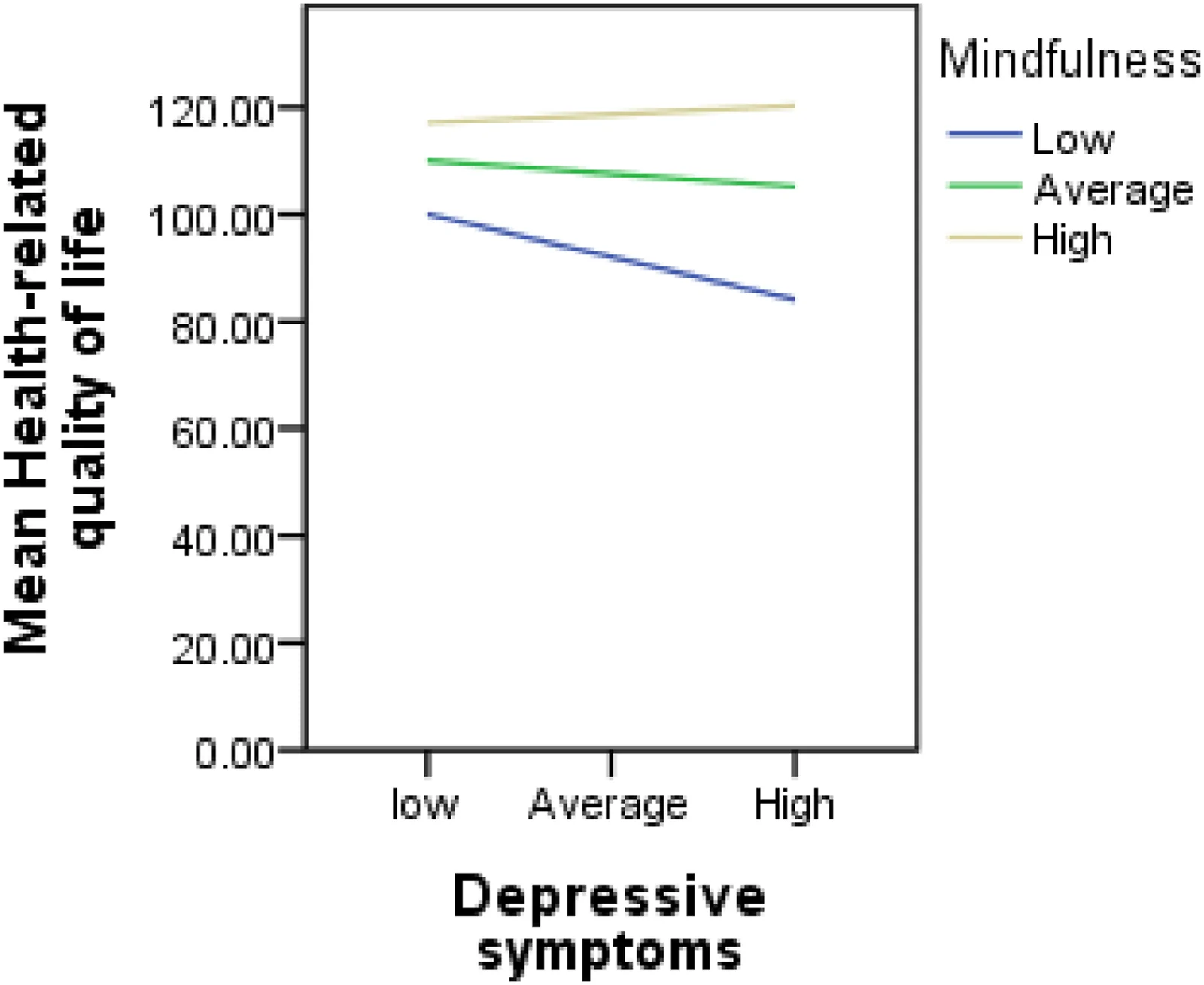
Showing the moderating role of mindfulness in the association of depression and health-related quality of life.

## Discussion

The current study found that the average scores of HRQoL (Mean = 104.69, SD = 29.93) was lower to those in prior studies among HIV patients (M = 302.81, SD = 61.06; Onu et al., 2021); (Mean = 114, SD = 32.01; Hoang et al., 2024) which suggests that professional counselling and psychological interventions are needed to help HIV patients to improve their HRQoL. Furthermore, the average score of mindfulness (Mean = 61.77, SD = 11.52) was lower than those of previous research among patients with Tuberculosis (Mean = 71.39, SD = 5.88; Al-Ghabeesh et al., 2024) but slightly higher than other researches among schizophrenia patients (Mean = 46.26, SD = 11.37; Bergmann et al., 2021). Regarding death anxiety, participants from the current sample on average scored slightly higher (Mean = 52.68, SD = 20.24) to those of other studies among patients with Tuberculosis (Mean = 48.30, SD = 6.88; Al-Ghabeesh et al., 2024). The average score on depression in this study (Mean = 20.57, SD = 8.55) was similar to those of prior research among schizophrenia patients (Mean = 18.30, SD = 7.55; Bergmann et al., 2021).

In support of our hypothesis, we found a significant interaction effect of mindfulness on death anxiety and HRQoL, such that death anxiety was associated with poor HRQoL among HIV patients with low mindfulness, but not those with average and high levels of mindfulness. In other words, higher levels of mindfulness protect HIV patients from the negative psychological impacts of death anxiety and improve their HRQoL. This finding is in agreement with previous research revealing that mindfulness prominently buffered or moderated the relationship between anxiety symptoms and psychological health (Nadeem & Koschmann, 2023; Shen et al., 2023), affirming that mindfulness is a very attractive psychosocial resource with strong capacity to mitigate the negative impact of stressors and promote mental health, consistent with the aforementioned theory: Mindful Coping Model (Garland et al., 2011). Among PLWH, death anxiety could involve a psychological condition wherein individuals consistently engaged in mortality-related thoughts, agitations and worries which are overwhelming and distressing, due to their HIV diagnosis and how best to cope with the disease (e.g., “I frequently think of my own death”; “It annoys me to hear about death”, “I think I am more afraid of death than most people”) (Tomás-Sábado et al., 2005). These thought patterns could cause patients to become very defensive, making it possible for the individual to utilise avoidance behaviour as a way of coping (Park & Pyszczynski, 2019). Such individual patients may potentially experience poor HRQoL (Sherman et al., 2010; Zhao et al., 2022). Although the diagnosis of HIV and the awareness of the protracted nature of its treatment is truly one existential crisis that is capable of provoking unpleasant feelings of uncertainty and increased thoughts of mortality, our findings have revealed that HIV patients who have higher levels of mindfulness are able to navigate through these existential problems and cope adequately with their health condition (Xiang-Zi & Jia-Yuan, 2023).

Being mindful involves greater ability to be aware of one’s inner and outer experiences without judging them as good or bad rather with acceptance attitude (Nadeem et al., 2023). Death mindfulness or awareness of death thoughts helps to change ones attitude to life by cultivating a sense of meaning in life and the acceptance of death as an inevitable end of every human being (Askarizadeh et al., 2022). Indeed, we need to re-echo the fact that death is certainly an inevitable end of every human being, and that people with greater mindfulness are mindfully aware of the inevitability of death but do not amplify, exaggerate or allow it to affect their daily functioning (Askarizadeh et al., 2022). Thus, when individual are confronted with a death-remembering condition such as HIV, a flexible cognitive-emotional strategy such as positive reappraisal that emanate from mindfulness, not only enhance the search for new experience and meaning in life (Aliche, 2023a; Garland et al., 2011), but also help to change the individual’s worldview by decreasing the person’s defensive responses and increasing death neutral acceptance (Park & Pyszczynski, 2019). This helps to improve compassion and kindness to oneself that essentially facilitate personal recovery (Askarizadeh et al., 2022; Park & Pyszczynski, 2019). Crucially, higher mindfulness could be an effective coping strategy for HIV patient by helping to reduce unhealthy thoughts and attitudes towards death (Askarizadeh et al., 2022). More so, a mindful awareness of the finiteness and fragility of life generally makes individual to focus more on achieving valued oriented goals, and engaging in healthier living with calmness and acceptance of every life situation (Xiang-Zi & Jia-Yuan, 2023). This kind of understanding and flexibility helps to improve HRQoL of HIV patients (Aliche et al., 2024; Gao et al., 2024; Gonzalez-Garcia et al., 2014). Therefore, the cultivation of mindfulness through mindfulness training could be an effective clinical approach to alleviating death related anxiety and improving HRQoL of PLWH.

Furthermore, the present study found a significant moderating effect of mindfulness in the association of depressive symptoms and HRQoL of HIV patients. Specifically, higher depressive symptom was associated with poor HRQoL only among HIV patients with low levels of mindfulness, but not those with average and high levels of mindfulness. This indicates that higher mindfulness protects HIV patient from the negative effect of depression on health outcome, consistent with previous findings (Hicks et al., 2018; Xu et al., 2018). Mindfulness offers HIV patients the opportunity to focus on the present moment, becoming aware of all feelings inside and outside the body and treating these feelings with a truthful, non-judgmental, and open mind (Burnett-Zeigler et al., 2023). Higher levels of mindfulness ameliorate depressive symptoms by enabling patients to disengage from negative emotions and depressogenic thinking pattern, much so, that patients are better able to take a more proactive approach towards addressing their health problems such as improving their health behaviour, and greater willingness to adhere to antiretroviral treatment (Musa et al., 2020).

As a conscious awareness, mindfulness promotes attention and adaptive cognitive functioning; preventing the occurrences of rumination and feelings of hopelessness and helplessness that characterised depressed patients (Burnett-Zeigler et al., 2023). This is made possible due to the inherent capacity of HIV patients with greater mindfulness abilities to easily regulate their emotions and utilise adaptive coping skills that helps to promote psychological wellbeing (Parmentier et al., 2019). Additionally, being mindful can serve as an important psychological resource enabling people to observe their feelings and thoughts in the moment without judging them as good or bad, providing valuable insight for mindfulness-based prevention and interventions for depressive symptoms among PLWH (Musa et al., 2021; Samhkaniyan et al., 2015). Therefore, it is important to re-emphasize that mindfulness facilitates adaptive functioning and positive emotional life by ameliorating HIV-related depressive symptoms and improving HRQoL of PLWH (Gonzalez-Garcia et al., 2014).

The findings of this study should be considered in the light of a few limitations. First, the use of self report measures and cross sectional design which could potentially preclude the assessment of causal inference. Second, Samples were drawn from a single tertiary health care institution in South-east Nigeria. Caution should be exercised when generalising the findings to other populations and those with different chronic disease. Third, the researchers were unable to obtain information on certain clinical characteristics of the HIV patients including the viral load, CD4 count among others which may impact on health outcome (Gonzalez-Garcia et al., 2014). In addition, we examined the effect of overall death anxiety score on the global HRQoL scores without observing their various dimensions. Different subtests of death anxiety might have differential effect on various domains of HRQoL of PLWH.

## Conclusion

This study explored the role of mindfulness in the association between death anxiety, depressive symptoms and HRQoL among PLWH. Although previous studies have explored the relationship between death anxiety and HRQoL, results of these studies have been insistent. The findings of the current research have contributed to literature by highlighting the protective role of mindfulness in buffering the negative psychological impact of death anxiety on HRQoL among PLWH. Moreover, this study also extended prior research by revealing that a greater ability to focus on what is going on here and now with a non-judgemental acceptance (mindfulness) moderated the effect of depressive symptoms on HRQoL among HIV patients. These findings underscore the importance of integrating clinical psychologists in the holistic treatment and care of PLWH. Such clinical efforts geared towards promoting HRQoL of HIV patients should begin with a careful evaluation of the patients’ levels of dispositional mindfulness. Those who may be identified as having low levels of mindfulness may benefit from a mindfulness-based therapy which could show some promise in protecting HIV patients from death-related anxiety and depressive symptoms, and consequently improve patients’ HRQoL and recovery.
